# Biodegradable Collagen Implants in Trabeculectomy

**DOI:** 10.5005/jp-journals-10008-1179

**Published:** 2015-01-15

**Authors:** Parul Ichhpujani, Tanuj Dada, Shibal Bhartiya

**Affiliations:** Assistant Professor, Glaucoma Services, Government Medical College and Hospital Chandigarh, India; Professor, Glaucoma Services, Dr Rajendra Prasad Centre for Ophthalmic Sciences, All India Institute of Medical Sciences, New Delhi, India; Senior Consultant, Glaucoma Services, Fortis Memorial Research Institute Gurgaon, Haryana, India

**Keywords:** Collagen implants, Subconjunctival fibrosis, Trabeculectomy.

## Abstract

Subconjunctival and subscleral fibrosis are the major causes of failure of filtering surgery. Antiproliferative agents have been successfully used to improve the long-term success of this surgery. Recent advancement in the field of glaucoma surgery has been the use of bioengineered, biodegradable, porous collagen-glycosaminoglycan matrix implant in the subconjunctival and/or subscleral space to modify the wound-healing process and reduce scar formation, hence improving the surgical success without the need for anti-fibrotic agents.

Biodegradable, collagen implants have shown favorable results when used with deep sclerectomy. There have been variable results regarding the success of trabeculectomy when combined with these implants. These implants also decrease the dose of mitomycin C required with trabeculectomy and hence, decrease the side effect associated with these drugs.

The use of the biodegradable implants in glaucoma surgery is still evolving and further studies are needed to find the appropriate surgical technique, the ideal size and site of placement and determine their long-term impact on trabeculectomy outcomes and complications.

**How to cite this article:** Ichhpujani P, Dada T, Bhartiya S. Biodegradable Collagen Implants in Trabeculectomy. J Curr Glaucoma Pract 2015;9(1):24-27.

## INTRODUCTION

Trabeculectomy, the ‘traditional’ filtration surgery, aims at reduction of intraocular pressure (IOP) in medically refractory glaucoma patients, by way of shunting aqueous humor through to the canal of Schlemm by excising part of the trabecular meshwork exposing the canal of Schlemm.^1,2^ This surgery has contradictory concepts: prevention of wound healing around the surgical fistula and inhibition of fibrosis of Tenon capsule to sclera on one hand and normal tissue repair of the overlying delicate conjunctiva to maintain the functional and anatomic integrity of the bleb on the other.

Surgical trauma to conjunctival, episcleral and iris tissue leads to the leakage of plasma proteins into the filtration site, precipitation of the clotting cascade and complement activation. This cascade of wound healing and eventual scarring result in fibrosis of the bleb or obstruction of the drainage fistula, leading to bleb failure.^3^ Hence, the two most important locations related to the success of trabeculectomy are the subconjunctival and the subscleral flap spaces.^4^

A successful filtration surgery requires a delicate balance between maintaining the integrity of the eye and prevention of excess scarring. The antimetabolites, mitomycin C (MMC) and 5-fluorouracil (5-FU) are a double edged sword. They help to modify the fibrotic response to the surgical insult,^5,6^ but their use is associated with complications, such as hypotony, hypotony maculopathy, suprachoroidal hemorrhage, choroidal effusions, bleb leak, blebitis, bleb encapsulation, failure and endophthalmitis.^7,8^ Amniotic membrane transplantation and expanded polytetrafluoroethylene (Gore-Tex) implants have also been tried as adjunctive modifications to enhance and maintain the desired effect of trabeculec-tomy, but not with much success.^9,10^

The development of tissue engineering has offered a viable solution to prevent scar formation.^11,12^ Tissue engineering involves the combination of a polymer scaffold with a population of stem, progenitor, or precursor cells. Tissue growth is modelled to favor development of a particular structure and if the polymer scaffold used is biodegradable, it can result in the formation of structures, which are remarkably similar to normal tissue.^13^

When used in glaucoma surgery, the use of collagen-glycosaminoglycan copolymers leads to random and relatively loose reorganization of regenerating myofibroblasts, fibroblasts and the secreted extracellular collagen matrix, resulting in reduced scar formation.^14,15^ These implants offer a potential alternative to antifibrosis agents as they produce more loosely organized, yet more abundant bleb tissue than a bleb created without antimetabo-lites. Moreover, these biodegradable polymers degrade into nontoxic compounds in the body, thus avoiding the need for surgical removal.

This chapter throws light on the available collagen implants, their surgical applications, complications and outcome.

### Collagen Implants

The three-dimensional collagen matrix of the available implants has been designed to achieve three goals:

- Physiological environment to control cell ingrowth
- Stiffness to maintain the inner porous structure
- Preservation of the function of the reservoir without collapse during the period of wound contraction and physical weight on the scleral flap to prevent shallow anterior chambers.

Collagen implants studied so far for filtering surgery, include:

Ologen Implant, Version 1

Marketed initially as OculusGen (Aeon Astron Europe BV, Leiden, The Netherlands), it is a porous implant comprising > 90% lyophilised porcine collagen and < 10% lyophilized glycosaminoglycan with a pore size of 10 to 300 mm.

Ologen Implant, Version 2

Ologen (Aeon Astron Corporation, Taipei, Taiwan) (also named iGen) consists of > 90% lyophilized porcine atelocollagen and < 10% lyophilized porcine glycosaminoglycan. Atelocollagen is a highly purified pepsin-treated type I collagen. A collagen molecule has an amino acid sequence, known as a telopeptide, at both the N and C terminals, which confers most of the collagen’s anti-genicity. Atelocollagen obtained by pepsin treatment is low in immunogenicity, because it is free of telopeptides.^16^ Currently, it is available in two sizes, 6 mm in diameter with 2 mm of thickness and 12 mm in diameter with 1 mm of thickness, and is placed over the scleral flap before conjunctival closure.

### Indications

- Primary trabeculectomy
- Failed trabeculectomy
- Neovascular glaucoma
- Refractory glaucoma
- Severe conjunctival scarring following surgery.

### Contraindication

- Known allergic reaction to collagen.

### Surgical Technique

1. A fornix based conjunctival flap is created in an upper quadrant.
2. Careful monopolar cauterization is performed on the surface of the sclera.
3. A limbus-based triangular flap of two-thirds of the scleral thickness is dissected.
4. The sclerotomy of 1 × 1 mm is performed, followed by a peripheral iridectomy.
5. The scleral flap is then repositioned and fixed with one loose-fitting 10-0 nylon suture.
6. A cylindrical ologen implant is positioned on top of the scleral flap, to exert pressure on the scleral flap to avoid postoperative hypotonia.
7. Finally, the conjunctiva is closed with 8-0 vicryl suture.
8. The standard postoperative regimen of a topical antibiotic four times a day and dexamethasone four times a day in a preservative-free preparation is followed.

### Scientific Evidence So Far: Trabeculectomies and Phacotrabeculectomies

Animal Studies

Collagen matrices were implanted in the right eyes of 17 rabbits after trabeculectomy, while left eyes served as surgical controls.^17^ In the implanted group, the IOP continued to decrease to 55% below baseline at postoperative week 4 as the implant gradually degraded. In the control group, IOP returned to the preoperative level by postoperative week 3. On histologic examination, a prominent bleb was noted in the implanted group while the control group had scar formation and limited bleb formation.

In another study, trabeculectomy with subconjunc-tival Ologen implantation was carried out in 30 rabbit eyes, while 6 eyes underwent trabeculectomy without the implant to serve as a control group.^18^ In the implant group, the mean IOP was reduced by between 42% and 35% at postoperative weeks 2, 3 and 4, whereas the mean IOP in the control group was reduced by between only 12% and 2%. In the implant group, histology at postoperative week 4, showed random collagen deposition and microcyst formation in the bleb after the matrix had degraded completely while the control group had dense collagen deposition subconjunctivally.

Human Studies

In a prospective, randomized, medium-term pilot study conducted on 40 glaucoma patients, Papaconstantinou and coworkers, failed to show any IOP-lowering advantage of the Ologen. This pilot study indicated a tendency to a higher incidence of complications with the collagen implant.^19^

In another prospective randomized clinical trial with a 2-year follow-up, 40 glaucomatous eyes were assigned to trabeculectomy with MMC or Ologen.^20^ There was no significant difference in the postoperative behavior between the two groups, with a highly significant and stable IOP reduction and very few antiglaucoma medications throughout the 2-year follow-up.

Senthil et al compared the safety and efficacy of trabe-culectomy with Ologen implant versus trabeculectomy with MMC in 39 eyes of Asian Indian subjects with medically uncontrolled primary glaucoma and found that the success of trabeculectomy and complications were similar in both Ologen and MMC groups at the end of 6 months.^21^

Lee et al also found that trabeculectomy utilizing collagen matrix showed similar clinical results compared to the current traditional trabeculectomy. Filtering bleb function as evaluated on slit-lamp and ultrasound biomicroscopy revealed that Ologen did not induce any additional advantageous features.^22^

As regards the height of filtering blebs and the space-occupying effect of collagen matrix inside filtering blebs, studies show varied results. Some studies have shown that the collagen matrix takes up full space inside a filtering bleb,^17,18^ while others have found that the collagen matrix is formed in a relatively small volume within a filtering bleb and then it gradually disappears.^23^ In all the studies in the early postoperative stage, filtering blebs in the ologen group were more prominent because of the subconjunctival implant than in the MMC group.

Dada T described a novel approach to trabeculectomy using ologen implant under the subscleral flap and subconjunctival area to prevent fibrosis and failure of surgery.^24^ By placing the implant subsclerally, an additional advantage of lesser subscleral fibrosis might be obtained for better control of IOP.

Dada et al also analyzed 33 eyes of 24 patients with primary open-angle glaucoma who underwent fornix-based trabeculectomy with subconjunctival Ologen implant and MMC.^25^ Their study showed encouraging short-term results as the mean preoperative IOP, 34.06 ± 6.56 mm Hg, decreased to 12.54 ± 1.67 mm Hg at 12 months.

Narayanswamy et al studied the efficacy and safety of Ologen™ implant in phacotrabeculectomy surgeries in 60 patients with primary glaucoma.^26^ The overall percentage reduction in IOP was 13% in the Ologen group and 26% in the MMC group (p = 0.05). At one year, the overall performance of Ologen in combined phacotrabeculectomy was suboptimal compared to combined surgery with MMC. Eyes in the Ologen group required more frequent bleb needling procedures.

Johnson MS et al compared the outcomes between patients undergoing trabeculectomy with an Ex-PRESS mini glaucoma device using MMC to those undergoing trabeculectomy with a Ologen.^27^ At 12 months postoperatively, the mean IOP was 12.1 mm Hg for the MMC group, and 13.12 mm Hg for the Ologen group (p = 0.34) suggest that ologen provides similar rates of surgical success as MMC for patients undergoing a filtering procedure using an Ex-PRESS mini glaucoma device.

### Complications

None of the studies document any possible Ologen™ specific side effects, such as allergy or translocation of the implant.

In the study by Min et al, encapsulated blebs were generated at a rapid pace and in larger amounts compared with conventional trabeculectomy.^23^ Most encapsulated blebs were observed between second week and 1 month after surgery. It has been postulated that noncontractile collagen―producing fibroblasts play a major role in the process of encapsulated blebs. Ologen might affect the generation of encapsulated blebs because the ologen is biodegradable, which causes a number of fibroblasts inside to move into a bleb. Therefore, the authors suggested using Ologen soaked in a relatively high concentrated antimetabolites or use of antimetabolite eye drops during the early postoperative period, or injection of antimetabo-lites in a bleb during the postoperative 1 or 2 weeks, in order to minimize the generation of encapsulated blebs.

Recently, Dada et al reported a case of blebitis with scleral abscess in a young patient who had undergone a trabeculectomy with MMC and a subconjunctival Ologen implant.^28^ Meticulous conjunctival closure should be done at the end of trabeculectomy as exposed collagen can act as a nidus of infection.

Suture lysis maybe difficult in some cases with Olo-gen due to poor visualization through the collagen and conjunctiva.

Success rate of trabeculectomy with the Ologen implant has been found to be lower than or equivalent to that achieved with MMC. Further modifications of the ologen implant in terms of size, surgical technique, and also molecular structure will be needed to improve the functional outcome.

Five Year Review

The use of biodegradable implants in glaucoma surgery is still evolving. The best place, subconjunctival or sub-scleral or combined, to use these implants is not known presently. Results of studies involving the use of these implants in trabeculectomy will provide information regarding the best surgical approach.

Over the next 5 years long-term success and associated complications with the use of these implants will come up to support the use of these implants. Drug eluting biodegradable implants may come up in the next 5 years to help modify the wound healing after glaucoma surgery and increase the success of surgery.

### Key Issues

- The success of glaucoma surgery is limited by scarring over the filtering site leading to late failure.
- Pharmacological modulating agents, like mitomycin C and 5-fluorouracil, have increased the success of surgery in patients at the cost of increased risk of late-bleb related sight threatening complications.
- Biodegradable implants have come up to modulate wound healing in glaucoma surgery and replace the use of anti-fibrotic agents.
